# High-density immobilization of a ginsenoside-transforming β-glucosidase for enhanced food-grade production of minor ginsenosides

**DOI:** 10.1007/s00253-019-09951-4

**Published:** 2019-07-09

**Authors:** Chang-hao Cui, Byeong-Min Jeon, Yaoyao Fu, Wan-Taek Im, Sun-Chang Kim

**Affiliations:** 1 Intelligent Synthetic Biology Center, 291 Daehak-Ro, Yuseong-Gu, Daejeon, 305-701 Korea; 2 The Key Laboratory of Biotechnology for Medicinal Plant of Jiangsu Province, Jiangsu Normal University, No. 101 Shanghai Road, Xuzhou, Jiangsu 221116 People’s Republic of China; 3 Department of Biological Sciences, Korea Advanced Institute of Science and Technology, 291 Daehak-Ro, Yuseong-Gu, Daejeon, 305-701 Korea; 4 Department of Biological Sciences, Hankyong National University, 327 Chungang-Ro, Anseong City, Kyonggi-Do 456-749 Korea; 5 KAIST Institute for Biocentury, Korea Advanced Institute of Science and Technology, 291 Daehak-Ro, Yuseong-Gu, Daejeon, 305-701 Korea

**Keywords:** Compound K, Ginsenoside F_1_, Deglycosylation, Biotransformation, *Corynebacterium glutamicum*

## Abstract

Use of recombinant glycosidases is a promising approach for the production of minor ginsenosides, e.g., Compound K (CK) and F_1_, which have potential applications in the food industry. However, application of these recombinant enzymes for food-grade preparation of minor ginsenosides are limited by the lack of suitable expression hosts and low productivity. In this study, *Corynebacterium glutamicum* ATCC13032*,* a GRAS strain that has been used extensively for the industrial-grade production of additives for foodstuffs, was employed to express a novel β-glucosidase (MT619) from *Microbacterium testaceum* ATCC 15829 with high ginsenoside-transforming activity. A cellulose-binding module was additionally fused to the N-terminus of MT619 for immobilization on cellulose, which is an abundant and safe material. Via one-step immobilization, the fusion protein in cell lysates was efficiently immobilized on regenerated amorphous cellulose at a high density (maximum 984 mg/g cellulose), increasing the enzyme concentration by 286-fold. The concentrated and immobilized enzyme showed strong conversion activities against protopanaxadiol- and protopanaxatriol-type ginsenosides for the production of CK and F_1_. Using gram-scale ginseng extracts as substrates, the immobilized enzyme produced 7.59 g/L CK and 9.42 g/L F_1_ in 24 h. To the best of our knowledge, these are the highest reported product concentrations of CK and F_1_, and this is the first time that a recombinant enzyme has been immobilized on cellulose for the preparation of minor ginsenosides. This safe, convenient, and efficient production method could also be effectively exploited in the preparation of food-processing recombinant enzymes in the pharmaceutical, functional food, and cosmetics industries.

**Electronic supplementary material:**

The online version of this article (10.1007/s00253-019-09951-4) contains supplementary material, which is available to authorized users.

## Introduction

Ginseng has been widely used to treat various diseases in East Asia for more than a thousand years and is increasingly used in foods and dietary supplements worldwide (Chung et al. 2011; Shi et al. 2013). Ginsenosides, triterpene saponins that are almost exclusively found in ginseng, are considered as the main active ingredients responsible for various pharmacological activities (Kim et al. 2013; Qi et al. 2010; Song et al. 2014). Six kinds of major ginsenosides (Re, Rg_1_, Rb_1_, Rb_2_, Rc, and Rd) normally constitute more than 90% of the total ginsenosides in various parts of ginseng (Shi et al. 2013; Zhou et al. 2014). Most of the minor ginsenosides, which have fewer sugar moieties attached on aglycon, are absent or are present in smaller amounts than those of the major ginsenosides (Figure S1) (Shi et al. 2013; Zhou et al. 2014). Many minor ginsenosides have higher chemical reactivities than those of abundant major ginsenosides in raw materials (Smith et al. 2014; Wong et al. 2015). Among these minor ginsenosides, Compound K (CK) and F_1_ show important pharmacological effects, including anti-inflammatory (Wang et al. 2015), anticancer (Cho et al. 2009; Hu et al. 2012), neuroprotective (Lee et al. 2013), anti-diabetic (Gu et al. 2013), and skin-healing effects (Lee et al. 2003; Lee 2018), supporting their potential application in the food and pharmaceutical industries.

However, CK and F_1_ are rare minor ginsenosides with low polarities and are absent or comprise less than 0.005% of raw ginseng or red ginseng (Park et al. 2013; Shi et al. 2013; Zhou et al. 2014). Thus, purifying large quantities of pure CK and F_1_ from the raw plant is extremely difficult and costly, resulting in a bottleneck in food applications. Microbial or enzymatic methods have been explored for CK and F_1_ preparation (Cui et al. 2016; Kim et al. 2011; Park et al. 2010). However, these methods exhibit low selectivity and poor productivity. As an alternative to preparing pharmacologically active CK and F_1_, recombinant enzymatic conversion methods have been explored to efficiently and specifically convert abundant major ginsenosides (Shin and Oh 2016).

Several recombinant glycosidases produce these two minor ginsenosides in gram-scale quantities from abundant major ginsenosides (An et al. 2017; Cui et al. 2013). Unfortunately, *Escherichia coli* is not a suitable expression host for preparing food-grade minor ginsenosides because it is not generally recognized as food- grade (GRAS) bacteria and also has the potential of generating endotoxins (Liu et al. 2016). Two recent studies have demonstrated the preparation of minor ginsenosides F_2_ and Rh_2_-Mix from major ginsenosides using expression systems of GRAS host strains (Li et al. 2016a; Siddiqi et al. 2017). Nevertheless, CK or F_1_ production has not been achieved using recombinant enzymes from GRAS hosts.

Furthermore, the production efficiencies of ginsenoside CK and F_1_ by recombinant enzymes also require improvement. Enzyme modifications based on protein structure have been conducted to increase productivity, but limited improvements have been achieved (Shin et al. 2017). This issue can be addressed by modifying downstream processes, such as increments in the enzyme concentration, which could accelerate the reaction to reduce the precipitates of intermediates in minor ginsenosides preparation and reduce the reaction volume or time.

Conventional enzyme concentration methods, e.g., heating evaporation, freeze-drying, ultrafiltration, and chromatographic and precipitation separation, are limited by their high-energy consumption, long time processes, and activity damages (Shire et al. 2004). The adsorption method using a cellulose-binding module (CBM) to immobilize recombinant enzymes on cellulose is an emerging technique with many important advantages, such as low-energy consumption, simple operation, high affinity of binding, and minimum enzyme damage (Li et al. 2016b; Oliveira et al. 2015; Yu et al. 2017). Furthermore, the supporting material (cellulose) is an abundant and safe material that is broadly applied as a food additive (Eichhorn et al. 2009; Je et al. 2017). This immobilization method exhibits a high protein-binding capacity using regenerated amorphous cellulose (RAC), which can be simply made by phosphoric acid treatment (Hong et al. 2008; You and Zhang 2013).

In this study, a food-grade expression and immobilization method was developed to meet the demand for the enhanced production of CK and F_1_. For this purpose, we selected MT619 among 10 glycosidase candidates with high activity for transforming protopanaxadiol (PPD)-type ginsenoside mixtures (PPDGM) or protopanaxatriol (PPT)-type ginsenoside mixtures (PPTGM) into CK and F_1_, respectively. The enzymatic properties and substrate specificities of MT619 were investigated. The enzyme was fused with CBM (C3a) from *Clostridium thermocellum* and expressed as a recombinant enzyme (C3a-MT619) in *Corynebacterium glutamicum* ATCC13032 for immobilization on RAC with a high protein density. The treatment of PPDGM and PPTGM with the immobilized enzyme yielded gram-scale CK and F_1_ with high efficiency. These findings represent a substantial advance over the efficient production of minor ginsenosides using recombinant enzymes owing to the lack of studies of ginsenoside-transforming glycosidases immobilized on cellulose.

## Materials and methods

### Chemicals and reagents

Standards of various ginsenosides (Rg_1_, Re, Rb_1_, Rc, and Rd) used in the present study were purchased from Hongjiu Co., Ltd. (Dalian, China). GypXVII, GypLXXV, Rg_3_(*S*), Rh_2_(*S*), F_2_, Compound K (CK), PPD, Rg_2_(*S*), Rh_1_(*S*), and PPT were prepared as described in our previous studies (Cui et al. 2014; Cui et al. 2017; Cui et al. 2013; Du et al. 2014). The other chemical reagents used were at minimum of extra pure grade.

### Synthesis and cloning of candidate glycosidases

The bacterial strains and vectors employed for the expression of MT619 and C3a-MT619 are shown in Table S1. As codon preference differs among taxa, the 10 glucosidase genes were re-translated into DNA favoring the codon usage of *C. glutamicum* ATCC13032 to enhance expression in this organism and synthesized and cloned into pGEX4T-1 by Mutagenex Co. (Suwanee, GA, USA). The sequence for the codon-optimized *mt619* gene was deposited into GenBank under accession number MK575514.

### Construction of MT619 and C3a-MT619 expression vectors

DNA fragments of interest were amplified by polymerase chain reaction (PCR) with *Pfu* polymerase (Elpis-Biotech, Daejeon, Korea) and purified using gel-extraction kits obtained from Enzynomics Co. Ltd. (Seoul, Korea). Primers used in the present study are listed in Table S2. Five fragments were amplified and gel-purified: pEX backbone (P3 and P4), pH 36 backbone (P8 and P9), c3a (P5 and P6), mt619-1 (P1 and P2), c3a-mt619 (P10 and P11), and mt619-2 (P2 and P3). These fragments were joined by Gibson assembly (New England Biolabs, Ipswich, MA, USA) to form pEX-mt619 (pEX backbone and mt619-1), pEX-c3a-mt619 (pEX backbone, c3a, and mt619-2), and pH36-C3a-MT619 (pH 36 backbone and c3a-mt619). The resulting plasmids were transformed into *C. glutamicum* ATCC13032 by electro-transformation using the method described previously (Ruan et al. 2015).

### Characterization of MT619

The protein concentrations in samples were determined using the Bradford reagent (Sigma, St. Louis, MO, USA) and the specific activity was determined using pNP-β-d-glucopyranoside (PNPGlc) as a substitute substrate at 37 °C. The reactions were monitored using a microplate reader Bio-Rad Model 680 (Bio-Rad, Hercules, CA, USA) with kinetic mode, and the release of p-nitrophenol was measured at 405 nm every 5 s. The effect and stability of pH and temperature, and metals and substrate preference on the enzymatic activity were determined as described previously (Cui et al. 2017).

Substrate preference of recombinant MT619 was determined using p-nitrophenyl (PNP) and o-nitrophenyl (ONP) glycosides as substrates (all from Sigma) at 37 °C. The following substrates were examined: PNP-β-d-glucopyranoside, PNP-β-d-galactopyranoside, PNP-β-d-fucopyranoside, PNP-N-acetyl-β-d-glucosaminide, PNP-β-l-arabinopyranoside, PNP-β-d-mannopyranoside, PNP-β-d-xylopyranoside, PNP-α-d-glucopyranoside, PNP-α-l-arabinofuranoside, PNP-α-l-arabinopyranoside, PNP-α-l-rhamnopyranoside, PNP-α-d-mannopyranoside, PNP-α-d-xylopyranoside, ONP-β-d-glucopyranoside, ONP-β-d-galactopyranoside, ONP-β-d-fucopyranoside, and ONP-α-d-galactopyranoside (all from Sigma).

### Biotransformation of the major ginsenosides using recombinant MT619

Purified MT619 was used to examine its hydrolyzing-specificity to the sugar moieties attached to PPT-type ginsenosides (Re and Rg_1_) and PPD-type ginsenosides (Rb_1_, Rd). The purified MT619 was reacted with Re, Rg_1_, Rb_1_, or Rd (2.0 mg/mL, pH 7.0) in a shaking incubator at 37 °C. The ginsenosides in samples were extracted by butanol and were identified by thin-layer chromatography (TLC).

### RAC preparation

RAC was prepared based on the method described previously (Hong et al. 2008). Briefly, 10 g of microcrystalline cellulose (SigmaCell 20) and 30 mL of distilled water were mixed to form a suspension. Two hundred milliliters of ice-cold 86% H_3_PO_4_ was carefully added to the mixture with stirring. After the cellulose solution turned transparent, it was placed on ice for one hour. Then, 800 mL ice-cold water was added with vigorous stirring by the addition of 200 mL at a time. The suspension mixture was centrifuged at 4000*g* and 4 °C for 20 min, and the supernatant was discarded. The cellulose pellet was washed four times with cold water to remove phosphoric acid. After neutralization using 2 M Na_2_CO_3_, the pellet was washed twice with water. The prepared RAC was stored at 4 °C as a 4-g RAC/L suspension with 0.2% sodium azide.

### Adsorption of C3a-MT619 on RAC

To estimate the binding capacity of C3a-MT619 attached to the RAC, adsorption isotherm measurements were taken. A sequence of tubes containing 0.1–10 mL of C3a-MT619 cell lysate (pH 7.0) with a fixed concentration (0.5 mg/mL) was prepared. To each tube, 0.4 mg of RAC was added and was incubated with shaking at 200 rpm for 10 min at 25 °C. The enzyme-immobilized RAC was centrifuged at 10,000 *g* for 5 min and the precipitate was washed twice with 1 mL of phosphate buffer (50 mM, pH 7.0). The obtained cellulose was assayed for β-glucosidase activity adsorbed on the cellulose. The maximum enzyme adsorption capacity (*A*_max_) of RAC was calculated by the Langmuir equation, as previously described (Hong et al. 2007). The *W*_max_ and *K*_p_ values were also calculated by mathematical methods (Bothwell and Walker 1995). The following equation was applied:

$$ {E}_{\mathrm{a}}=\frac{W_{\mathrm{max}}{K}_{\mathrm{p}}{E}_{\mathrm{f}}}{1+{K}_{\mathrm{p}}{E}_{\mathrm{f}}} $$

*E*_a_ indicates adsorbed C3a-MT619 (mg/g RAC), *W*_max_ indicates the maximum C3a-MT619 adsorption per liter (mg/g RAC), and *E*_f_ indicates free C3a-MT619 (mg/g RAC).

### Preparation of recombinant MT619 from *C. glutamicum* ATCC13032

For fed-batch cultivation and to obtain a high cell density of recombinant enzymes, defined and semidefined media supplemented with kanamycin (30 mg/L) were used to cultivate *C. glutamicum* ATCC13032 harboring pH36-C3a-MT619 in a 10-L stirred-tank reactor (Fermentec Co., Chungju, Korea) with a 6-L working volume at 400 rpm. As a seed culture, *C. glutamicum* ATCC13032 harboring pH36-C3a-MT619 was inoculated into 200 mL of defined medium containing 20 g/L glucose in a 1-L baffle flask and incubated at 30 °C for 20 h at 200 rpm. The medium consisted of 10 g of (NH_4_)_2_SO_4_, 3 g of K_2_HPO_4_, 1 g of KH_2_PO_4_, 2 g of urea, 2 g of MgSO_4_, 200 μg of biotin, 5 mg of thiamine, 10 mg of calcium pantothenate, 10 mg of FeSO_4_, 1 mg of MnSO_4_, 1 mg of ZnSO_4_, 200 μg of CuSO_4_, and 10 mg of CaCl_2_ per liter with 25 mg/L kanamycin.

The protein was constitutively expressed by the H36 promoter, as described previously (Yim et al. 2014). After the cell density reached an OD_600_ of 100, the cells were harvested by centrifugation at 4000*g* for 20 min. The pellets were suspended in 50 mM sodium phosphate buffer (pH 7.0); then, the cells were broken by sonication (Branson Digital Sonifier, Mexico City, Mexico). The cell debris was removed via centrifugation at 4000*g* for 20 min. The C3a-MT619 in cell lysate was absorbed by RAC with shaking for 15 min at 25 °C.

### Optimization of the substrate concentration

CK and F_1_ production were evaluated using PPDGM and PPTGM. To determine the optimal concentration of substrates for the biotransformation reaction, enzyme-immobilized RAC was mixed with an equal volume of substrates at concentrations of 10–100 mg/mL at 37 °C. Samples were withdrawn at regular intervals and analyzed by HPLC.

### Scale-up CK and F_1_ production

The scaled-up transformation was performed in a shaking incubator at 200 rpm and 37 °C. The reaction started with 20 mg/mL and 75 mg/mL substrates (PPDGM or PPTGM) as a final concentration by addition of immobilized C3a-MT619. Samples were analyzed by HPLC to determine the product concentrations of ginsenosides CK and F_1_.

### High-performance liquid chromatography analysis

The HPLC analysis of samples in the present study was performed using an Agilent 1260 Infinity HPLC system (Agilent Co., Santa Clara, CA, USA). Separation of ginsenosides was conducted on a YMC ODS C18 column (5 μm, 250 × 4.6 mm; YMC, Kyoto, Japan) with a guard column (Eclipse XDB C18, 5 μm, 12.5 × 4.6 mm; Agilent Technologies, CA, USA). The gradient elution system consisted of water (A) and acetonitrile (B) using the following gradient program: 0–8 min, 32% B; 8–12 min, 32–65% B; 12–15 min, 65–100% B; 15–15.1 min, 100% B; 15.1–25 min, 100–32% B; 25–26 min, 32% B. The detection wavelength was set to 203 nm at a flow rate of 1.0 mL/min.

### TLC analysis

TLC was conducted using 60F_254_ silica gel plates (Merck, Darmstadt, Germany) and CHCl_3_-CH_3_OH-H_2_O (65:35:10, *v*/*v*/*v*) as the developing solution. The results were visualized by 10% H_2_SO_4_ by heating at 110 °C for 5 min.

## Results

### Cloning of 10 glycosidase candidates for CK and F_1_ production

**Fig. 1:**
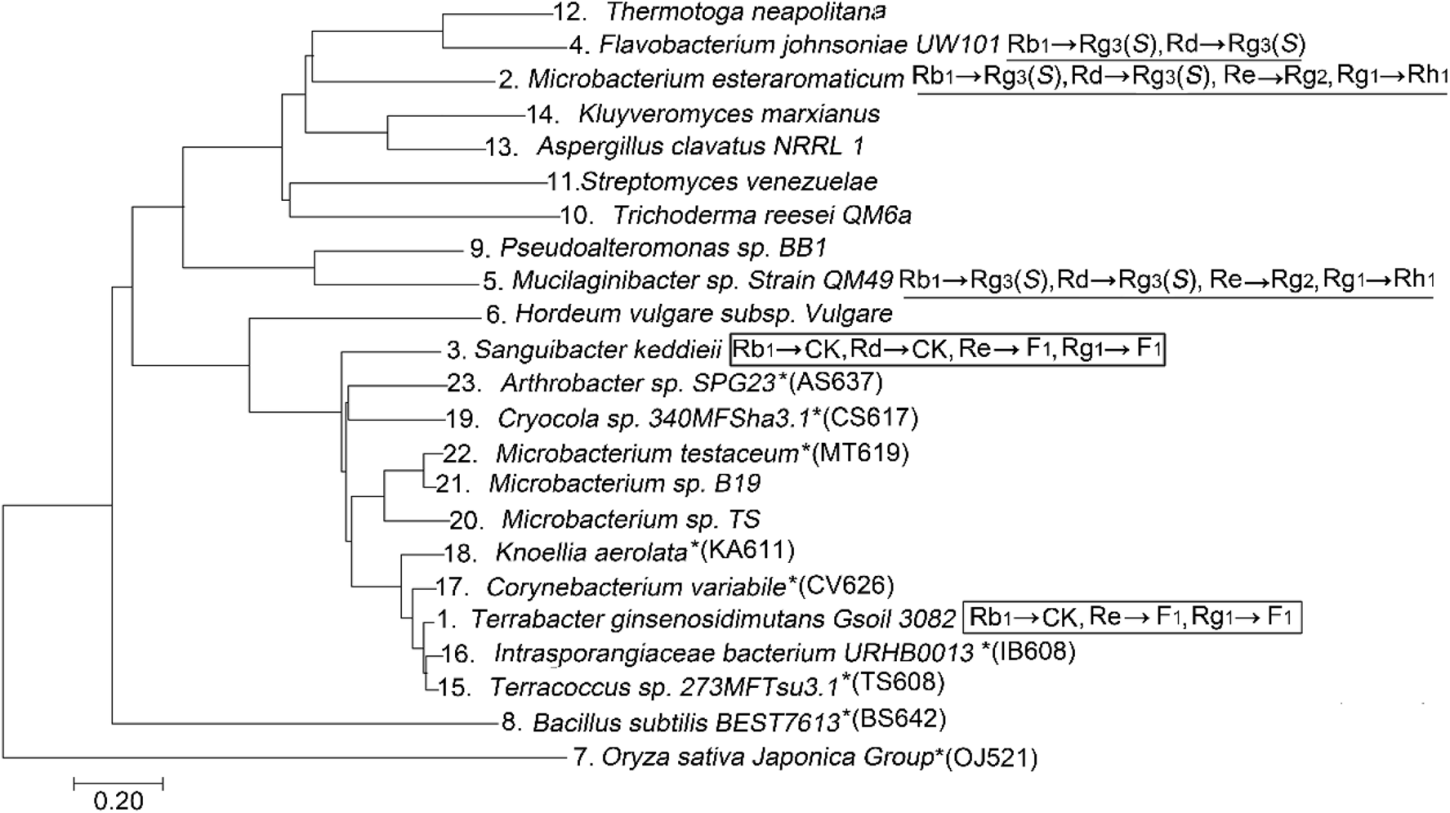
Evolutionary relationships among the characterized and candidate glycosidases in glycoside hydrolase family 3 (GH3). Amino acid sequences of these proteins were retrieved from the CAZy and NCBI/EMBL databases (accession numbers are indicated in Table 1). The evolution was assumed using the neighbor-joining method in MEGA7 program (Kumar and Stecher 2016). The bar at the bottom represents 20 amino acid substitutions per 100 residues

To identify highly productive enzymes, 19 uncharacterized family 3 glycosidases were chosen as candidates based on amino acid similarity by BLAST (blast.ncbi.nlm.nih.gov). In the phylogenetic tree (Zhang and Sun 2008), nine known enzymes that can transform the major ginsenosides Rb_1_ and Rg_1_ into CK and F_1_ or Rg_3_ and Rh_1_ formed separate groups (Fig. 1). The ginsenoside-transforming characteristics of glycosidases are related to the properties of their amino acid sequences. Among the candidates, ten glycosidases (Table 1), which grouped with enzymes producing CK and F_1_, were selected for heterologous expression and characterization. The coding sequences of glycosidases were codon-optimized for expression in *C. glutamicum* ATCC13032, synthesized, and cloned into a pGEX4T-1 vector. These synthesized enzymes were expressed in *E. coli* and determined by SDS-PAGE (Figure S2). All candidates were successfully expressed in *E. coli*, except OJ521, and seven showed glucosidase activity against pNP-β-glucopyranoside (PNPGlu), not including AS637 and BS642 (Table 1). Their ginsenoside-transforming activities were examined using Rb_1_, Re, and Rg_1_ as substrates. Interestingly, the enzymes with CK and F_1_ producing abilities were grouped separately from Rh_2_ and Rh_1_ by the sequence-based phylogenetic tree. Based on TLC (Figure S3), all clones were able to hydrolyze Rg_1_ into F_1_, except BS642. Ginsenoside Re could be hydrolyzed by six glycosidases (TS608, IB608, CV626, CS617, and MS614) to PPT via F_1_. Six enzymes (TS608, IB608, CV626, KA611, CS617, and AS637) showed Rb1→GYP17→GYP75 transforming activity and TS608 and CS617 produced CK by hydrolyzing the C20 outer glucose of Gyp75. MS614 and MT619 produced F_2_ and CK that were different from other clones. The expression and activities of all clones are summarized in Table 1. These results also provide evidence for the amino acid sequence-activity relationships of ginsenoside-transforming glycosidases. We selected MT619, which exhibited the highest activity among all candidates, as a suitable enzyme for the production of CK and F_1_ from major ginsenosides.

**Table 1:** Cloned glycosidases and their characteristics

Name	Organism	Source	pNP-β-glucopyranoside activity	Rb_1_	Re	Rg_1_	GenBank accession no.
TS608	*Terracoccus* sp. 273MFTsu3.1	Bacteria	+	G75, CK	F_1_, PPT	F_1_	WP_020141869.1
IB608	*Intrasporangiaceae bacterium*	Bacteria	+	G17, G75	F_1_, PPT	F_1_	WP_026862722.1
CV626	*Corynebacterium variabile*	Bacteria	+	G17, G75	F_1_, PPT	F_1_	WP_030201081
KA611	*Knoellia aerolata*	Bacteria	+	G17, G75	−	F_1_	WP_035939434.1
CS617	*Cryocola* sp. 340MFSha3.1	Bacteria	+	G75, CK	F_1_, PPT	F_1_	WP_020076619.1
AS637	*Arthrobacter* sp. SPG23	Bacteria	Weak	G75	−	F_1_	WP_043484882.1
MS614	*Microbacterium* sp. TS-	Bacteria	+	F_2_, CK	F_1_, PPT	F_1_	WP_023951833.1
MT619	*Microbacterium testaceum*	Bacteria	+	F_2_, CK	F_1_, PPT	F_1_, PPT	WP_013585536.1
BS642	*Bacillus subtilis* BEST7613	Bacteria	−	−	−	−	BAM49096.1
OJ521	*Oryza sativa* Japonica Group	Plant	−	−	PPT	−	AAN01354.1

### Expression of C3a-MT619 in *C. glutamicum* ATCC13032

**Fig. 2:**
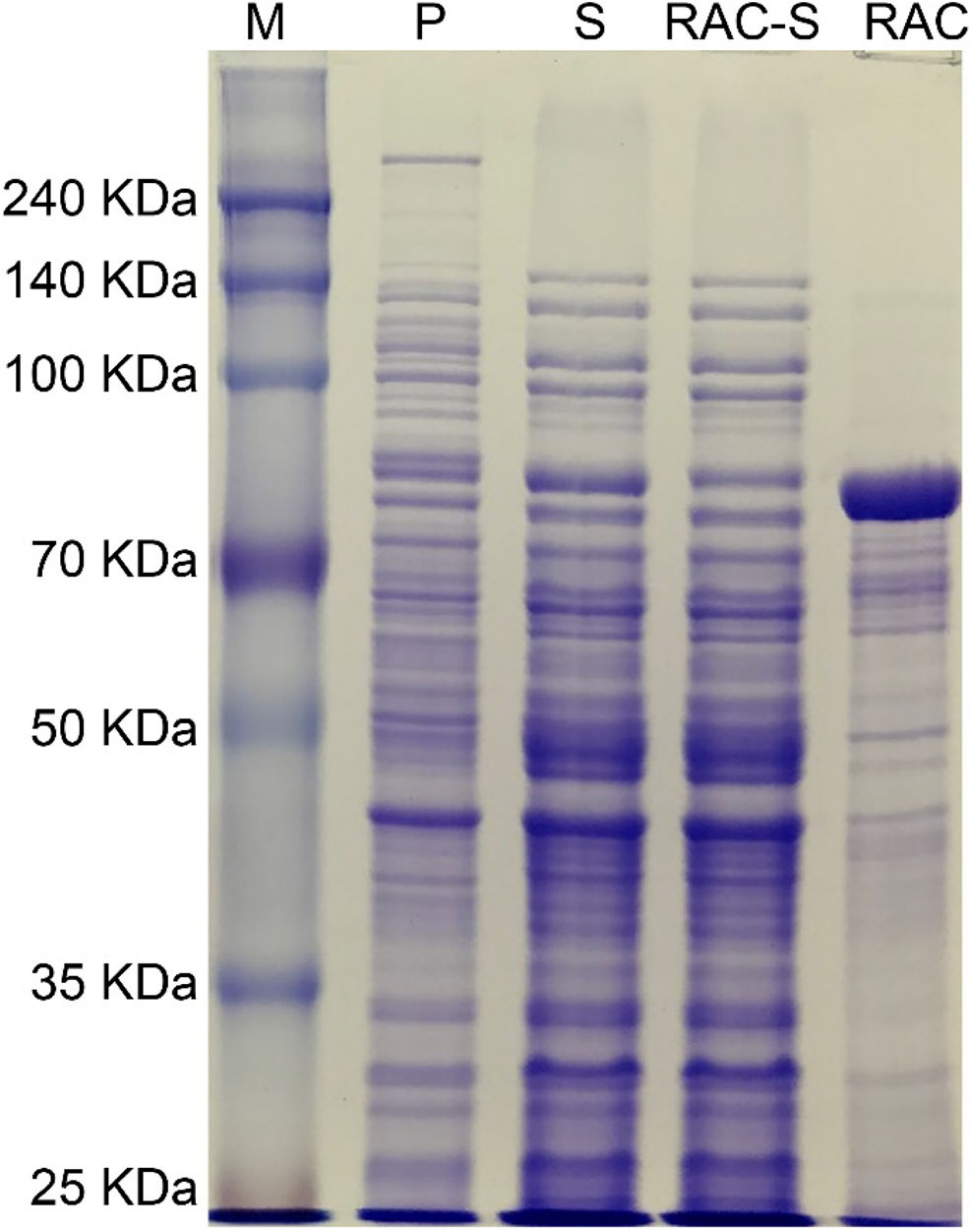
SDS-PAGE analysis of the *C. glutamicum* ATCC13032 cell extracts containing the recombinant proteins and RAC pull-down proteins. Lane M, protein marker; Lanes S, soluble cell extract containing C3a-MT619; Lanes P, precipitant of the cell extract, respectively; Lane RAC-S, the supernatant after immobilization; Lane RAC, RAC adsorbed C3a-MT619

For high-density immobilization, a CBM (C3a) from *C. thermocellum* (Oliveira et al. 2015), was engineered at the N terminus of MT619 to construct C3a-MT619 with a flexible linker (G4S)_2_ to connect the two modules. The *mt619* and *c3a-mt619* genes consist of 1878 and 2376 bps and encode 625 and 791 amino acids, with molecular weights of 68.3 and 86.3 kDa. The genes were amplified by PCR and subcloned into the pEX4T-1 and pH 36 vectors for heterologous expression in *E. coli* BL21 and *C. glutamicum* ATCC13032. C3a-MT619 was expressed continuously in *C. glutamicum* ATCC13032 by the promoter H36, which is efficient for heterologous expression (Yim et al. 2014). After cultivation for 24 h, significant expression bands with the predicted molecular masses were observed (Fig. 2). Recombinant C3a-MT619 contains 1.41 ± 0.03% of the total proteins in the *C. glutamicum* ATCC13032 lysate, which is 21.5% of the expression level of the enzyme in *E. coli* BL21.

### Characterization of recombinant MT619

MT619 and C3a-MT619 proteins were purified by Ni column and DEAE column chromatography. The predicted molecular mass (68.3 and 86.3 kDa) of the native β-glucosidase was validated by SDS-PAGE (Figure S4). Based on the examination of activity using PNPGlu, the recombinant C3a-MT619 expressed in *C. glutamicum* ATCC13032 had an enzyme activity of 61.9% of the MT619 heterologously expressed in *E. coli*.

**Fig. 3:**
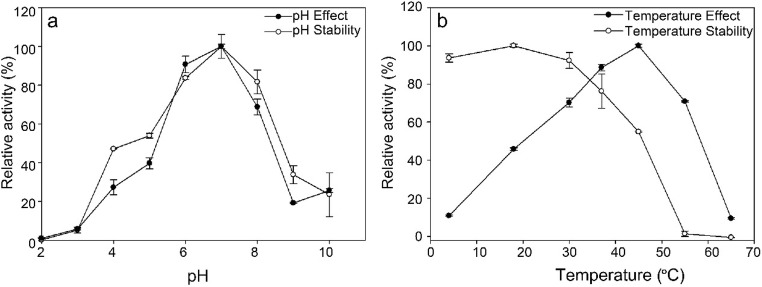
Effects of pH (**a**) and temperature (**b**) on the β-glucosidase activity and enzyme stability of MT619

The optimum pH and temperature were examined using purified MT619. The effect of pH on enzymatic activity was determined using 2.0 mM PNPGlc as a substrate. The pH stability of recombinant C3a-MT619 was determined by measuring enzymatic activity after incubation in each buffer at 4 °C for 12 h. MT619 was relatively stable from pH 6.0 to 8.0 and had optimal activity at pH 7.0; from pH 9.0, the enzyme activity decreased rapidly and at pH 5.0, the enzyme activity reduced to 54.0% (Fig. 3a). The effect of temperature on enzymatic activity was tested by incubating the enzyme with an optimal pH buffer containing 2.0 mM pNPGlc for 5 min. The optimal temperature for enzyme activity was 45 °C; at 30 °C and 37 °C, the enzyme had a relative activity of 70.3% and 88.6%, respectively. Recombinant MT619 was relatively stable at temperatures lower than 30 °C. 76.3% and 54.9% of enzyme activity were retained after incubation for 2 h at 37 °C and 45 °C, respectively, and no activity was detected above 55 °C (Fig. 3b). Though MT619 has the highest activity at 45 °C, the ginsenoside-transforming reaction was conducted at 37 °C to extend the stable transformation activity.

The effects of chemical reagents and metal ions on MT619 activity were determined using a concentration of 10 mM. The enzyme activity was not affected by β-mercaptoethanol, Ca^2+^, Na^+^, K^+^, and Mg^2+^; however, activity was lost in the presence of Cu^2+^, Hg^2+^, and Zn^2+^. EDTA did not affect its activity, indicating that divalent cations are not necessary for the enzyme activity. MT619 was significantly inhibited by Co^2+^ and Mn^2+^. No significant positive effects on the activity of the MT619 were found for the tested ions and agents (Table S3).

The glycoside specificity of MT619 was examined using PNP and ONP glycosides. MT619 was only active against the glucose moiety of PNP-β-d-glucopyranoside and ONP-β-d-glucopyranoside. It showed maximum activity towards PNP-β-d-glucopyranoside and 28.7% relative activity towards ONP-β-d-glucopyranoside.

### Ginsenoside-transformation activity of MT619

**Fig. 4:**
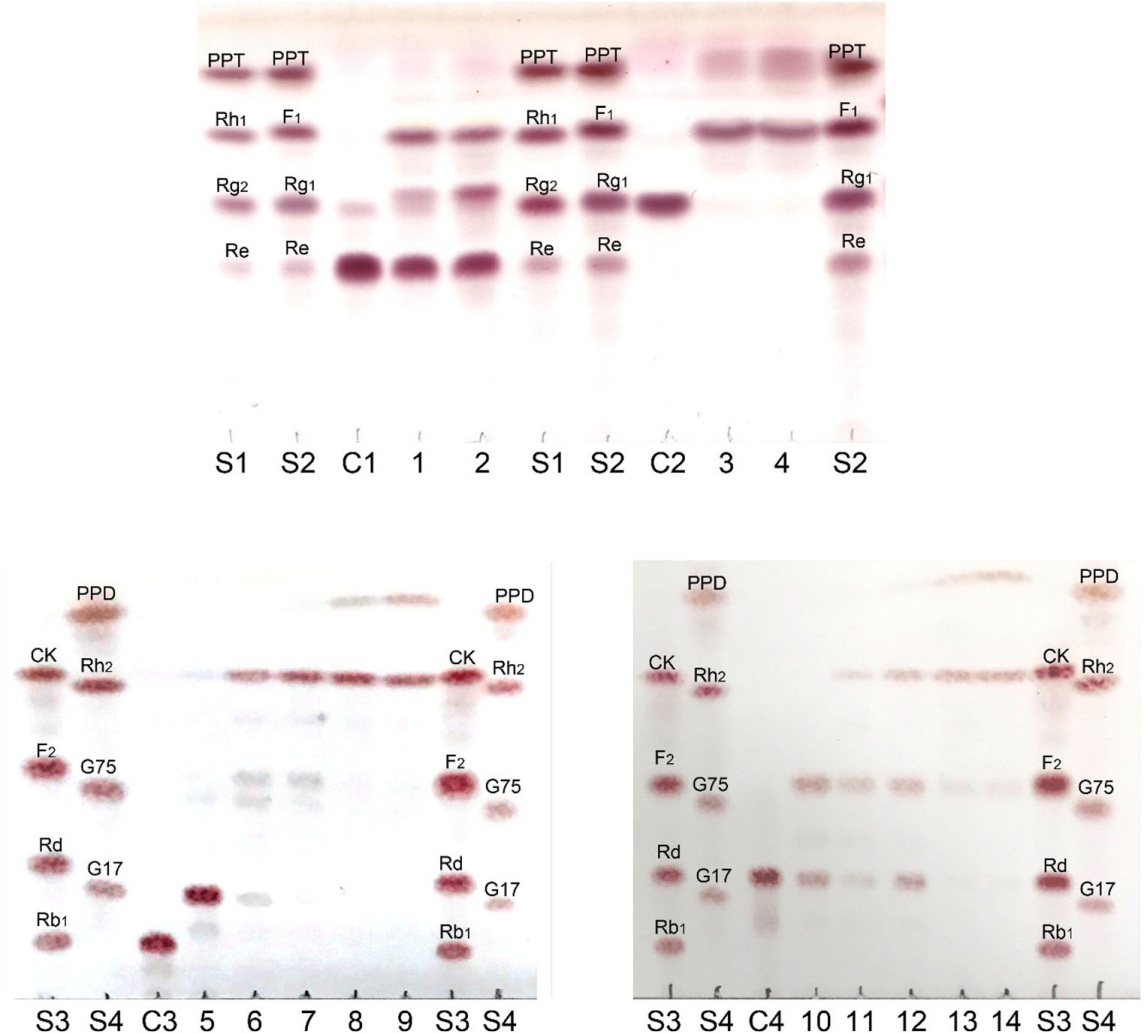
Biotransformation of Rb_1_, Rd, Re, and Rg_1_ by purified MT619 (0.1 mg/mL) analyzed by TLC. S1, S2, S3, and S4, ginsenoside standards; C1, substrate Re; 1 and 2, biotransformation of Re by purified MT619 for 2 h and 4 h, the suspected trans-glycosylated product-MT1 is indicated with an arrow; C2, substrate Rg1; 3 and 4, biotransformation of Rg1 by MT619 for 2 h and 4 h; C3, substrate Rb1; 5, 6, 7, 8, and 9, biotransformation of Rb1 by MT619 for 0.01 h, 1 h, 2 h, 6 h, 12 h, and 24 h; C4, substrate Rd; 10, 11, 12, 13, and 14, biotransformation of Rd by MT619 for 1 h, 2 h, 6 h, 12 h, and 24 h. MT1, the suspected transglycosylated product, is indicated by a blue arrow in 2

**Fig. 5:**
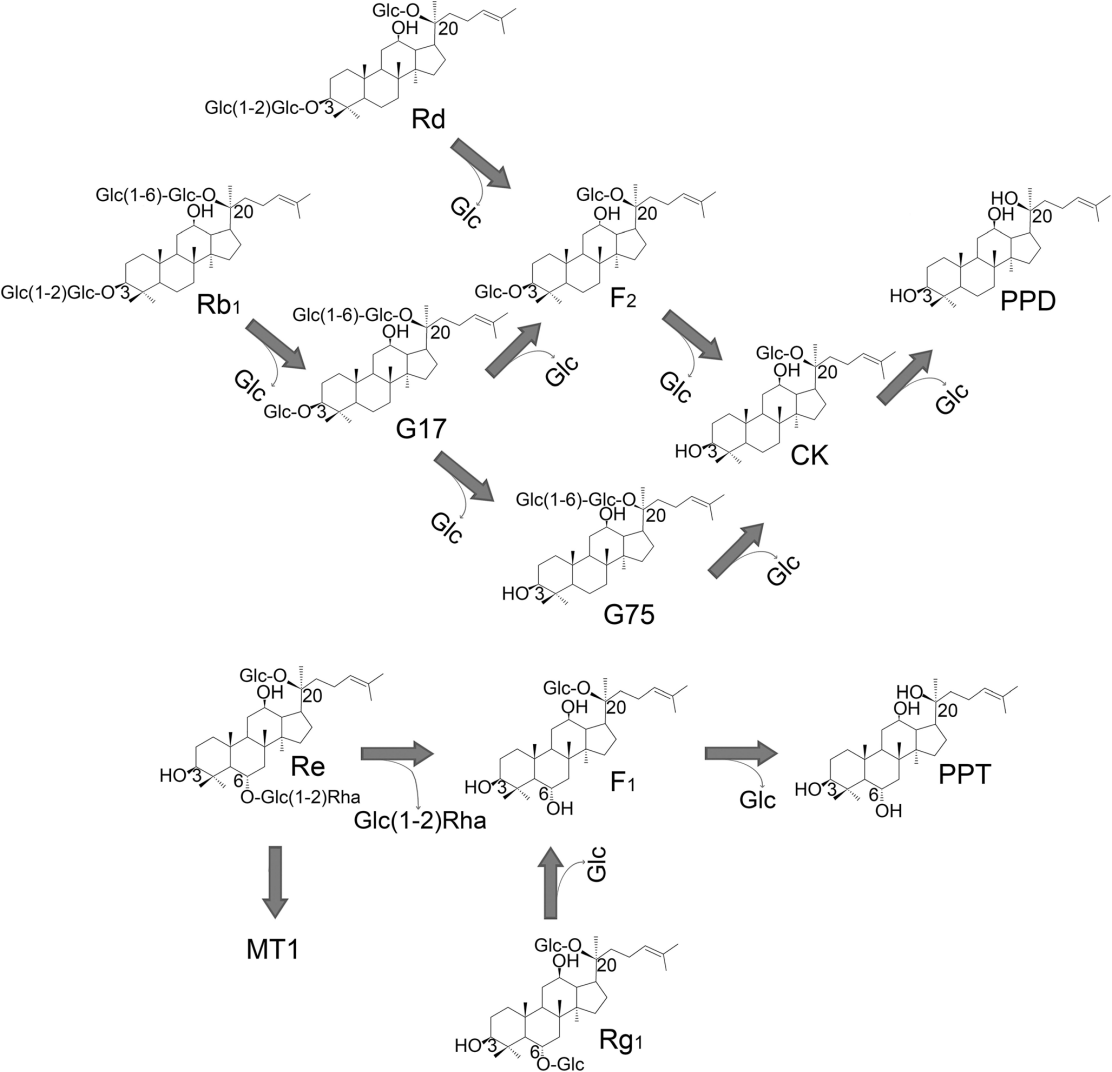
Transforming pathways of ginsenosides Rb_1_, Rd, Re, and Rg_1_ and their metabolites hydrolyzed by recombinant MT619

MT619 could clearly transform four major ginsenosides (Re, Rg_1_, Rb_1_, and Rd), as evidenced by the Rf values in a TLC analysis (Fig. 4). The proposed biotransformation pathways by MT619 for the PPD ginsenosides are Rb_1_→GypXVII(G17)→GypLXXV(G75) & F_2_→CK→PPD; Rd→F_2_→CK→PPD; Re→F_1_→PPT; Rg_1_→F_1_→PPT (Fig. 5). This enzyme has less hydrolytic activity against the glucose at the C20 position, which may result in the production of CK and F_1_ from the abundant higher polarity ginsenosides. The transforming pathways are similar to the reported ginsenoside-transforming glucosidases (An et al. 2010; Shin et al. 2017). However, MT619 showed the highest F_1_ production activity, which was 7.4 times higher than that of BgpA, used for the gram-scale production of this rare minor ginsenoside (An et al. 2017). Interestingly, we also found an unexpected band (MT1) whose Rf value was higher than those that of Rg_1_ and Rg_2_ (Fig. 4). This may represent the trans-glycosylated products of Re, produced by MT619, whose molecular weight is 784.7, as determined by MS/MS detection in positive electrospray ionization mode. The molecular structure of MT1 and the transglycosylation activity of MT619 would be analyzed in future studies.

### Adsorption of C3a-MT619 on RAC

**Fig. 6:**
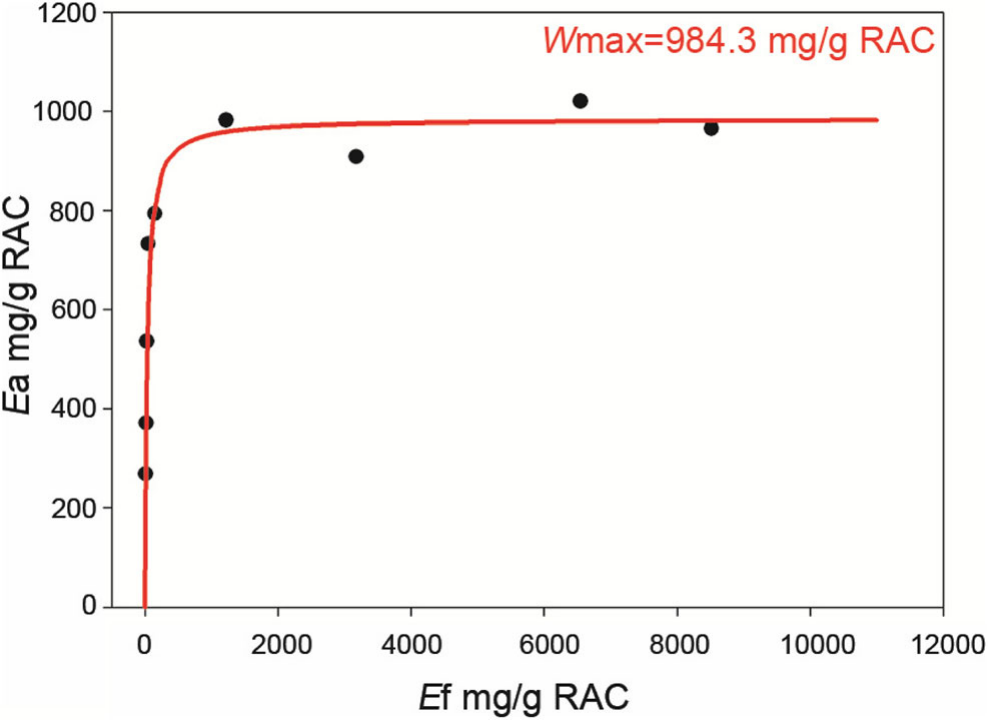
Various amounts of C3a-MT619 protein adsorption on a fixed amount of RAC at 25 °C. The Langmuir equation was used to calculate the *A*_max_ value

CBMs are often fused to the partner proteins to assist their production and purification (Oliveira et al. 2015). The expressed C3a-MT619 was adsorbed on the surface of RAC, which has a much larger external surface area than that of microcrystalline cellulose (Hong et al. 2008). The crude cell lysates of C3a-MT619 were obtained by sonication of the cell pellets and mixed with RAC; thus, C3a-MT619 was adsorbed on the surface of cellulosic material as revealed by SDS-PAGE analysis (Fig. 2). The adsorption curves of the immobilized C3a-MT619 enzyme detected by glucosidase activity obeyed the Langmuir isotherms, suggesting single layer adsorption (Fig. 6). The maximum adsorption capacity of the fusion enzyme was 984 mg/g RAC, and the maximum enzyme concentration on RAC is approximately 78.7 mg/mL RAC (condensed by centrifugation at 10,000*g* for 5 min), which is 286 times greater than the cell lysate concentration.

Scale-up absorption was conducted using 5000 mL of cell lysate and 50 mL of RAC. 66.0% of C3a-MT619 in the cell lysate was immobilized on RAC by shaking for 10 min, resulting in a 61.8-fold increase in concentration compared with the cell lysate. The immobilized concentration of C3a-MT619 on RAC was 217 mg/g RAC (17.3 mg/mL RAC, condensed by centrifugation at 10,000*g*, 5 min), and SDS-PAGE showed that some unspecific proteins in *C. glutamicum* ATCC13032 were co-immobilized on cellulose (Fig. 2); these were consistent with previous results (Li et al. 2016b). The immobilized C3a-MT619 retained over 78.0% of its activity after successive washing with 10 volumes of buffer repeated 10 times. Although the immobilization of C3a-MT619 was quite stably attached to RAC in this experiment, the re-usability of the immobilized enzymes is limited by the co-precipitation of the generated CK and F_1_.

### Optimization of substrate concentration for production of CK and F_1_

**Fig. 7:**
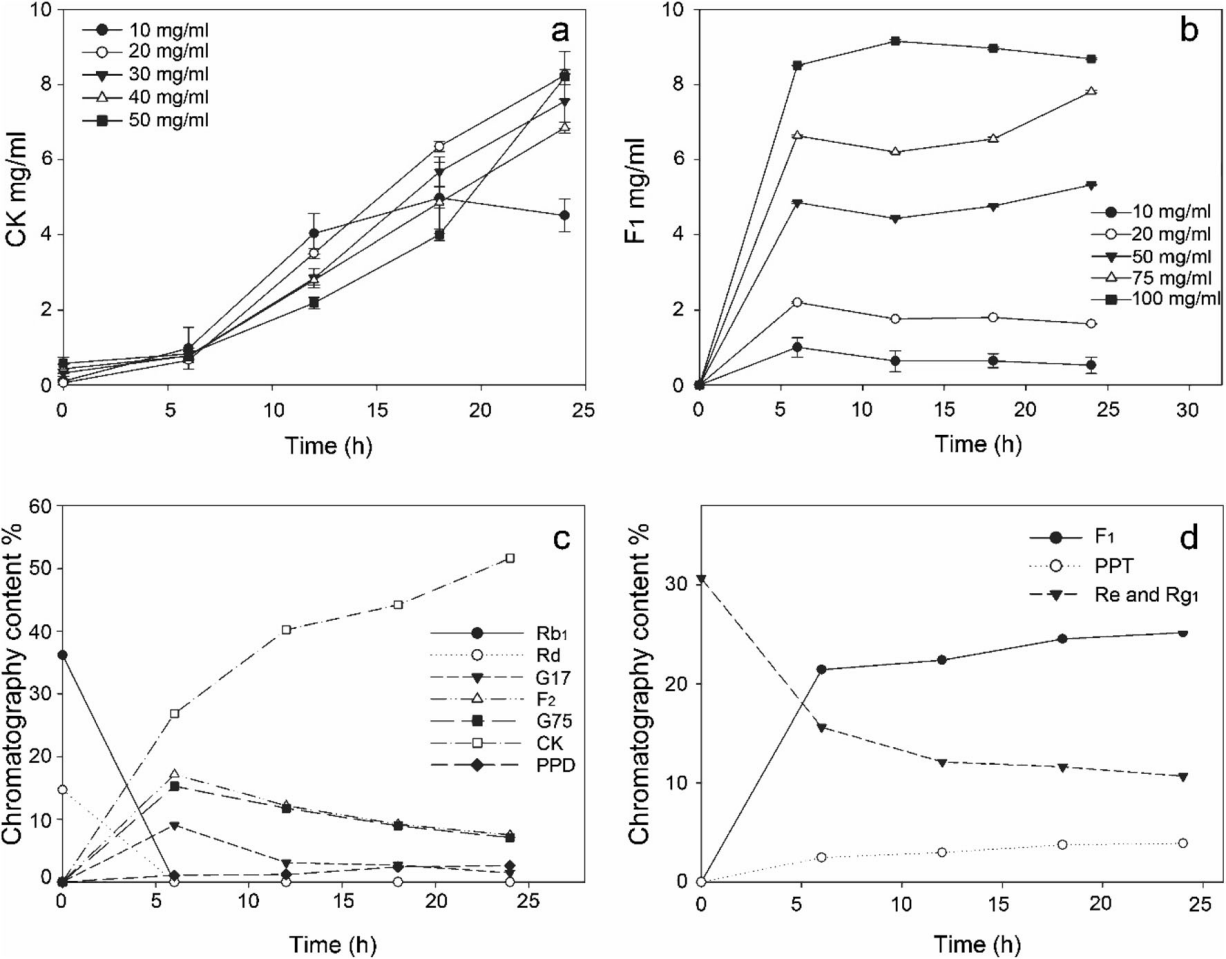
Effect of the substrate concentration on the production of CK **(a**) and F_1_ (**b**) using immobilized C3a-MT619, and scale-up productions (CK (**c**); F_1_ (**d**))

The enzyme reactions were performed using immobilized C3a-MT619 (217 mg/g RAC), and PPTGM and PPDGM were used as substrates owing to their relative abundance in crude ginseng extracts (Wan et al. 2008; Zhao et al. 2007). Various substrate concentrations of PPTGM and PPDGM (10–100 mg/mL) were examined to determine the appropriate reaction conditions for reduced reactor volumes and costs (Fig. 7a, b). The immobilized enzyme transformed 20 mg/mL PPDGM into CK with 8.25 mg/mL product concentration (Fig. 7a). PPDGM (10 mg/mL) reached its peak concentration after 18 h and decreased when CK (the final product) was transformed into PPD by C3a-MT619. Higher concentrations of PPDGM (> 20 mg/mL) reduced productivity. Though a substrate concentration of 50 mg/mL resulted in similar CK concentrations, we choose 20 mg/mL for the scaling-up the productivity. The concentration of F_1_ was increased in proportion to the substrate concentration, and the maximum product concentration of F_1_ was 9.14 mg/mL using 100 mg/mL PPTGM for 24 h (Fig. 7b). Owing to the advantages of using small quantities of enzymes and complete conversion of substrates, the PPDGM and PPTGM concentrations of 20 mg/mL and 75 mg/mL, respectively, were adopted for the scaled-up production of CK and F_1_.

### Mass production of CK and F_1_ using immobilized C3a-MT619

**Fig. 8:**
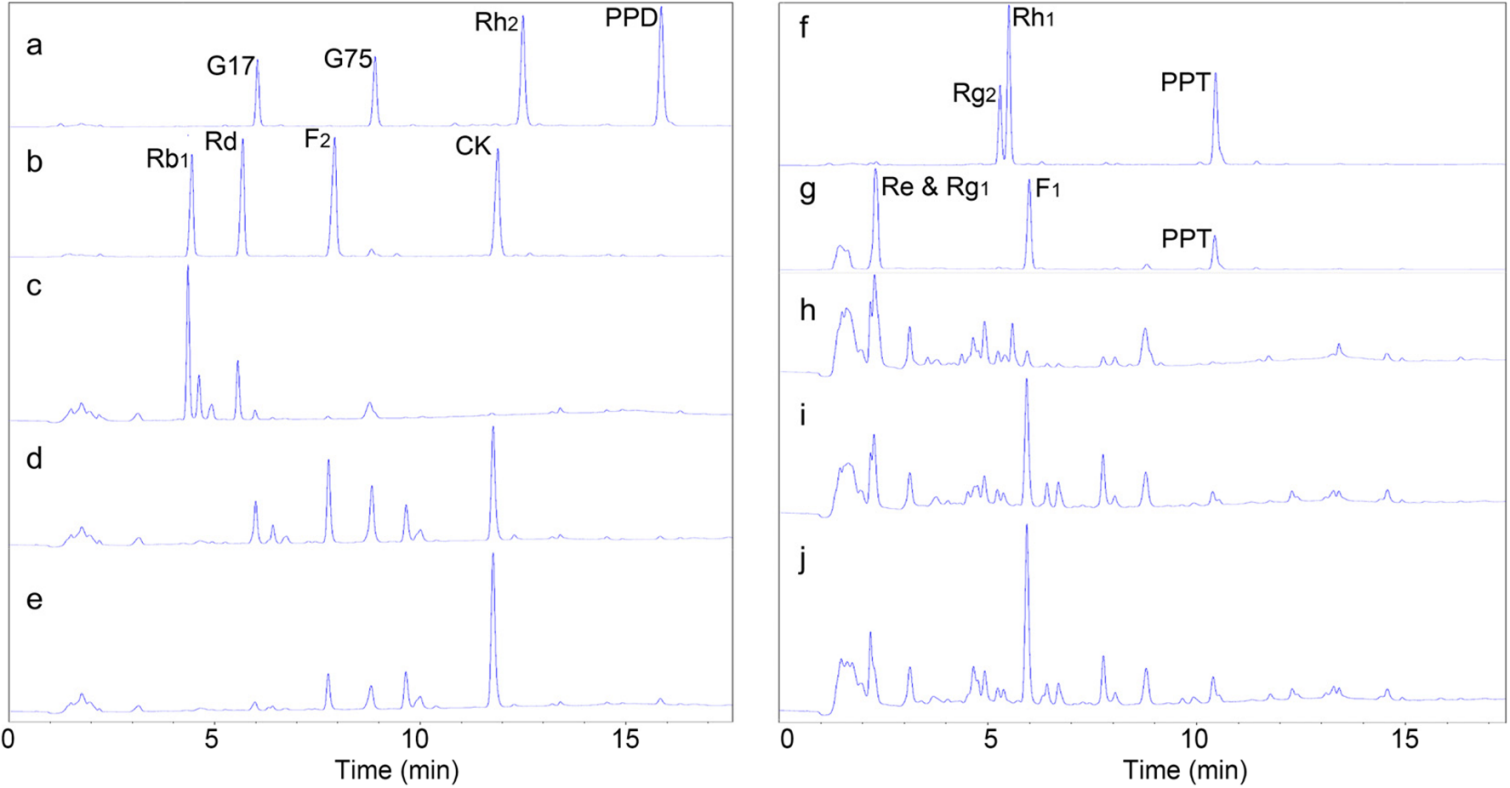
HPLC analysis of the transformation of ginsenosides using immobilized C3a-MT619. **a** Ginsenoside standards (G17, G75, Rh_2_, PPD). **b** Ginsenoside standards (Rb_1_, Rd, F_2_, CK). **c** PPDGM. **d** CK produced after 6 h of reaction of immobilized C3a-MT619 with PPDGM. **e** CK produced after 24 h of reaction. **f** Ginsenoside standards (Rg_2_, Rh_1_, PPT). **g** Ginsenoside standards (Re, Rg_1_, F_1_, PPT). **h** PPTGM. **i** F_1_ produced after 6 h of reaction of immobilized C3a-MT619 with PPTGM. **j** F_1_ produced after 24 h

Commercially available PPDGM and PPTGM were used as substrates for the mass production of CK and F_1_ by the immobilized enzymes with an enzyme-substrate volume ratio of 1:5 for 24 h (Fig. 7c, d). PPTGM (40 mL; 75 mg/mL) was used to produce ginsenoside F_1_ by the conversion of the immobilized C3a-MT619. A total of 54.3% of Rg_1_ and Re was transformed in 24 h with 9.42 g/L F_1_ product. The reaction for CK production consisted of 30 mL of C3a-MT619 immobilized RAC and 3 g of PPDGM (substrate, 20 mg/mL) in 150 mL of 50 mM sodium phosphate buffer. As shown in Fig. 7c, Rb_1_ and Rd in PPDGM were completely converted by the immobilized C3a-MT619 in 6 h. CK was produced at a concentration of 7.86 g/L in 24 h, with a 79.2% molar yield. Samples for CK and F_1_ production drawn and analyzed by HPLC are shown in Fig. 8. The produced CK and F_1_ were isolated from the reaction mixture using macroporous resins. Finally, 1.38 g and 1.59 g of CK and F_1_ products with 51.6% and 25.2% chromatographic purity were produced.

## Discussion

Recent studies have focused on the many pharmacological effects of ginsenoside CK and F_1_ owing to their potential food, cosmetics, and pharmaceutical applications. F_1_ is a potential drug for pigmentary disorders, atherosclerosis, and aging-related diseases (Hou et al. 2018; Lee et al. 2018; Qin et al. 2017). CK showed anticarcinogenic effects (Yang et al. 2015), outstanding skin anti-aging, anti-diabetes, and anti-arthritis effects (Chen et al. 2015; Lee et al. 2018; Wei et al. 2015). A human trial confirmed that CK is safe and well tolerated over the treatment period (Chen et al. 2017a), and pre-clinical experiments and early clinical trials have also been performed (Chen et al. 2017b; Zhou et al. 2018). However, the contents of minor ginsenosides in ginseng functional food products are low (less than 5.0%) (Ha et al. 2013; Sun et al. 2009).

Highly efficient and specific enzymatic transformation may be a promising method for minor ginsenoside preparation, especially using recombinant enzymes. In the last decade, the CK productivity from ginseng extracts increased to 4.2 mg/mL, which was accomplished using two recombinant glycosidases by Shin et al (Shin et al. 2016a). Similarly, F_1_ productivity increased to 6.7 mg/mL using a recombinant β-glucosidase from *Terrabacter ginsenosidimutans* Gsoil3082^T^ by our research group (An et al. 2017). However, both these studies used *E. coli* as host for enzyme preparation.

There is limited use of recombinant proteins from *E. coli* as food additives because *E. coli* is a non-GRAS species (Burdock and Carabin 2004). The use of *C. glutamicum* ATCC13032 has been suggested for application in production of food-grade material as it is an endotoxin-free GRAS strain, with many biotechnological advantages—easy gene manipulation, and growth rate, requirement of inexpensive media, and many industrial-grade food applications (Lv et al. 2012; Shin et al. 2016b; Yim et al. 2014). Hyaluronic acid and recombinant isomerase have been produced by recombinant *C. glutamicum* for applications in the food industry (Cheng et al. 2016; Shin et al. 2016b). However, the low expression levels of recombinant proteins in GRAS strains reduce their efficiency in the production processes.

One-step purification, concentration, and immobilization of enzymes using low-cost cellulosic materials would greatly facilitate enzyme preparation and decrease protein purification costs. Furthermore, cellulose has been used in various kinds of foods, such as traditional desserts, low cholesterol diets, vegetarian meats, and as additives, owing to their natural abundance, lack of toxicity, low cost, and intriguing mechanical properties (Eichhorn et al. 2009; Habibi et al. 2010; Je et al. 2017; Moon et al. 2011; Nishino et al. 2004; Wang et al. 2016; Youssef et al. 2016). This convenient and efficient immobilization method could also be usefully exploited in the preparation of food-processing recombinant enzymes in the functional food and cosmetic industries.

In conclusion, we describe the isolation of a ginsenoside-hydrolyzing β-glucosidase (MT619), its heterologous expression in *C. glutamicum* ATCC13032, concentration and immobilization on RAC by protein–cellulose interactions, and the efficient production of CK and F_1_. Compared with traditional CK and F_1_ preparation methods using the *E. coli* host system, the expression level is decreased, but this method of immobilization and production offers a number of advantages. (1) A GRAS strain was used for enzyme preparation, which is effective for food processing. (2) The immobilization, purification, and concentration of the enzyme is a simple, one-step process with high capacity for supporting materials. (3) The supporting material, cellulose, is an environmentally friendly, sustainable biomaterial and is widely used as a food additive. (4) The high density and purity of the immobilized enzymes increased productivity substantially. Thus, our findings demonstrate that the concentrated and immobilized β-glucosidase that was heterologously expressed in *C. glutamicum* can be used to efficiently produce CK and F_1_ from crude material from ginseng extracts. We believe that this method will provide an alternative approach for the food-grade production of CK and F_1_, as core functional biomaterials in the health food industries.

## Electronic supplementary material

ESM 1 (PDF 465 kb)
