# Supplementary material for: High-density immobilization of a ginsenoside-transforming β-glucosidase for enhanced food-grade production of minor ginsenosides
Source: Appl Microbiol Biotechnol. 2019 Jul 9;103(17):7003–15. doi: 10.1007/s00253-019-09951-4 (PMC6690934; doi:10.1007/s00253-019-09951-4)
Supplement: Supplementary file 1 — (PDF 465 kb) [file 253_2019_9951_MOESM1_ESM.pdf]

Supplementary Materials

Applied Microbiology and Biotechnology

High-density immobilization of a ginsenoside-transforming β-glucosidase for enhanced food-grade production of minor ginsenosides

Chang-hao Cui[1,2], Byeong-Min Jeon[3], Yaoyao Fu[2], Wan-Taek Im[4], and Sun-Chang Kim[1,3,5]

[1] Intelligent Synthetic Biology Center, 291 Daehak-Ro, Yuseong-Gu, Daejeon 305-701, Korea;

[2] The Key Laboratory of Biotechnology for Medicinal Plant of Jiangsu Province, Jiangsu Normal University, No.101 Shanghai Road, Xuzhou, Jiangsu 221116, P. R. China;

[3] Department of Biological Sciences, Korea Advanced Institute of Science and Technology, 291 Daehak-Ro, Yuseong-Gu, Daejeon 305-701, Korea;

[4] Department of Biological Sciences, Hankyong National University, 327 Chungang-Ro Anseong city, Kyonggi-Do 456-749, Korea

[5] KAIST Institute for Biocentury, Korea Advanced Institute of Science and Technology, 291 Daehak-Ro, Yuseong-Gu, Daejeon 305-701, Korea

Chang-hao Cui and Byeong-Min Jeon contributed equally to this work.

*Correspondence: sunkim@kaist.ac.kr; Tel: +82-42-350-4450; Fax: +82-42-350-2619.

Supplementary Figures

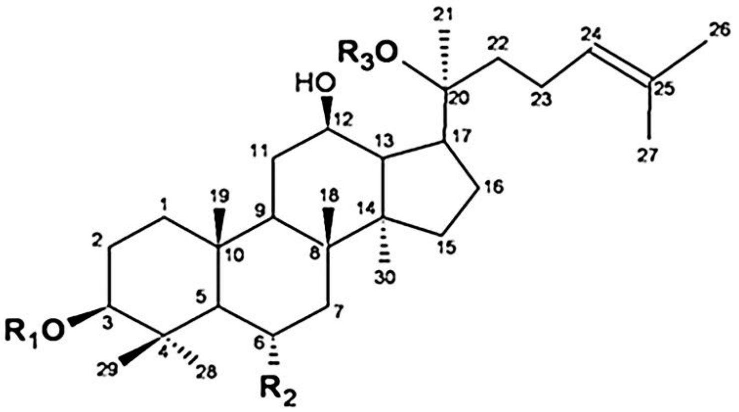

Ginsenoside	R_1	R_2	R_3	Types	Types
Rb_1	Glc(1→2)Glc-	H-	Glc(1→6)Glc-	Major	PPD type ginsenosides
Rb_2	Glc(1→2)Glc-	H-	Arap(1→6)Glc-	Major	
Rc	Glc(1→2)Glc-	H-	Araf (1→6)Glc-	Major	
Rd	Glc(1→2)Glc-	H-	Glc-	Major	
G17	Glc-	H-	Glc(1→6)Glc-	Minor	
F_2	Glc-	H-	Glc-	Minor	
Rg_3	Glc(1→2)Glc-	H-	H-	Minor	
G75	H-	H-	Glc(1→6)Glc-	Minor	
Rh_2	Glc-	H-	Glc-	Minor	
C-K	H-	H-	Glc-	Minor	
PPD	H-	H-	H-	Minor	
Re	H-	Rha(1→2)-Glc-O-	Glc-	Major	PPT type ginsenosides
Rg_1	H-	Glc-O-	Glc-	Major	
Rg_2	H-	Rha(1→2)-Glc-O-	H-	Minor	
Rh_1	H-	Glc-O-	H-	Minor	
F_1	H-	HO-	Glc-	Minor	
PPT	H-	HO-	H-	Minor	

Glc: glucopyranoside, Arap: arabinopyranoside, Araf: arabinofuranoside Xyl:xylopyranoside, Rha:rhamnopyranoside

Fig. S1 Chemical structures of protopanaxadiol ginsenosides.

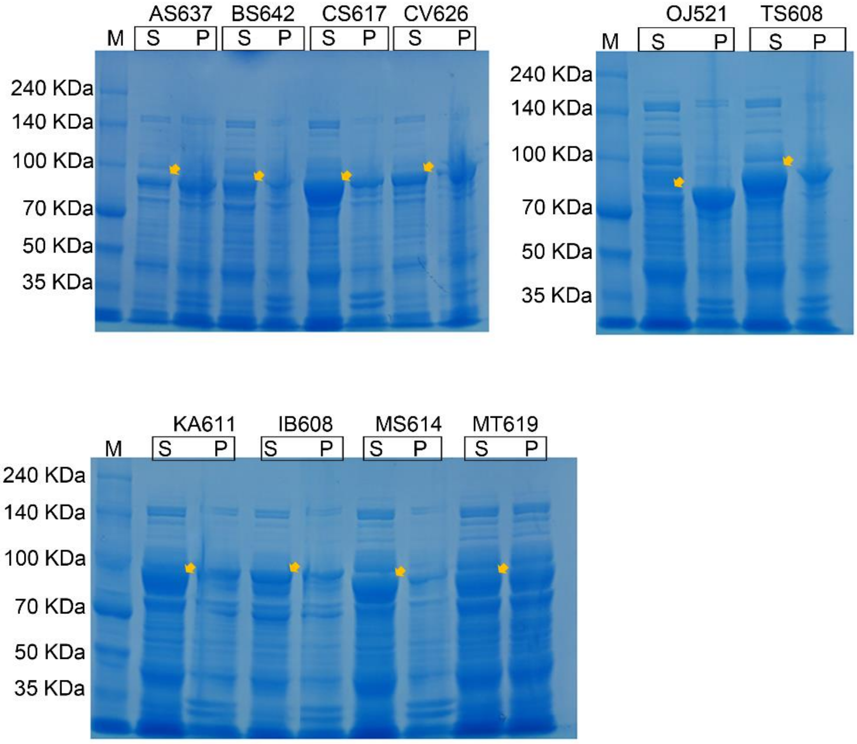

Fig. S2 SDS-PAGE analysis of the recombinant candidates expressed in *Escherichia coli*. Lanes: M, molecular weight standard; P, insoluble fraction of the crude extract of the induced recombinant BL 21 (DE3) cells; S, soluble fraction of the crude extract of the induced recombinant BL 21 (DE3)

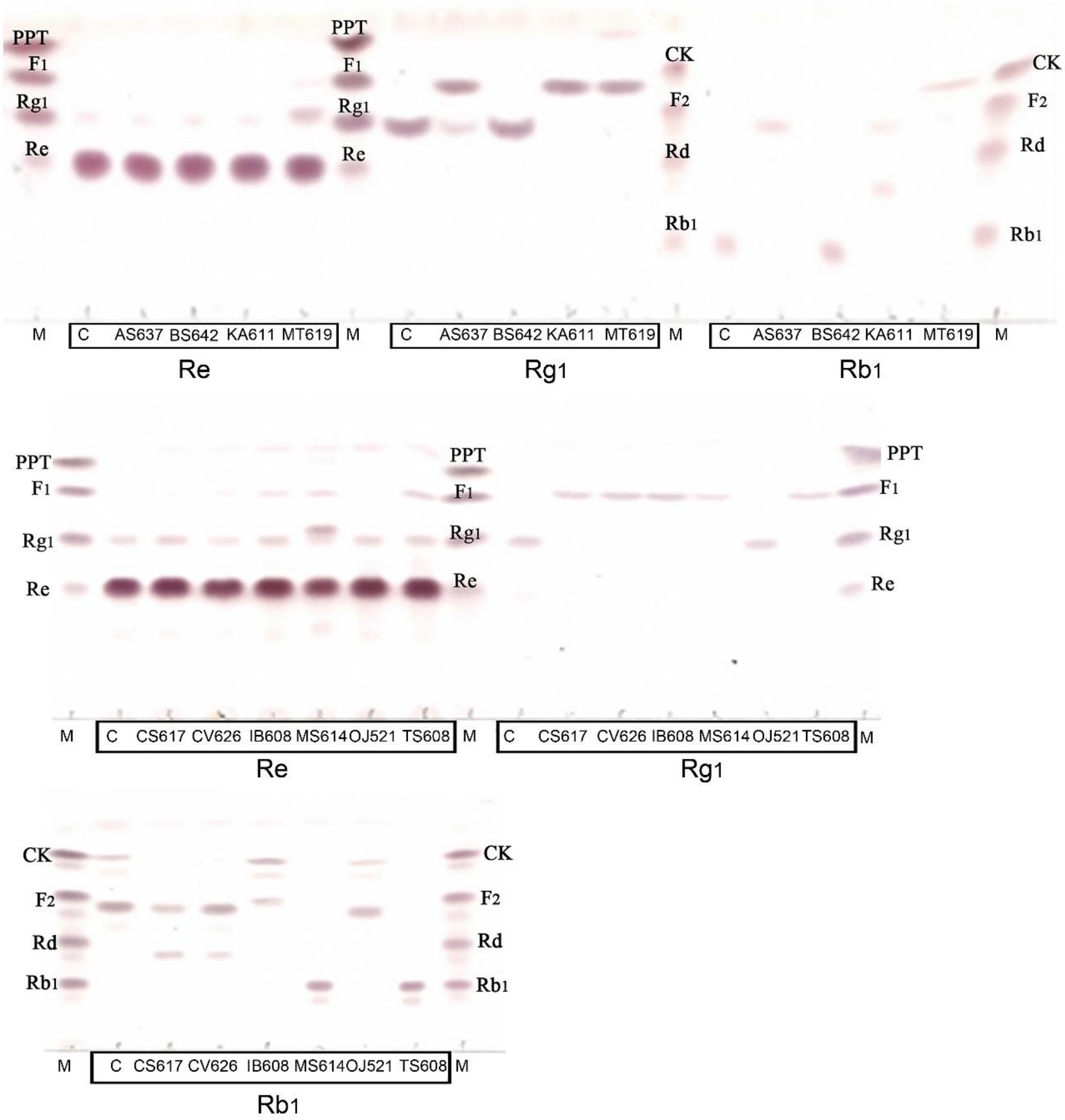

Fig. S3 Thin layer chromatography (TLC) analyses of biotransformation of Rb1, Re and Rg1 by recombinant candidates. The reaction time was 120min at a purfied enzyme concentration of 1.0 mg/ml in PBS buffer. Developing solvent: CHCl3-CH3OH-H2O (65:35:10, lower phase). Lanes: Lane C, substrate(1.0mg/ml), Lane M, ginsenoside standards (PPD-type ginsenoside mixtures).

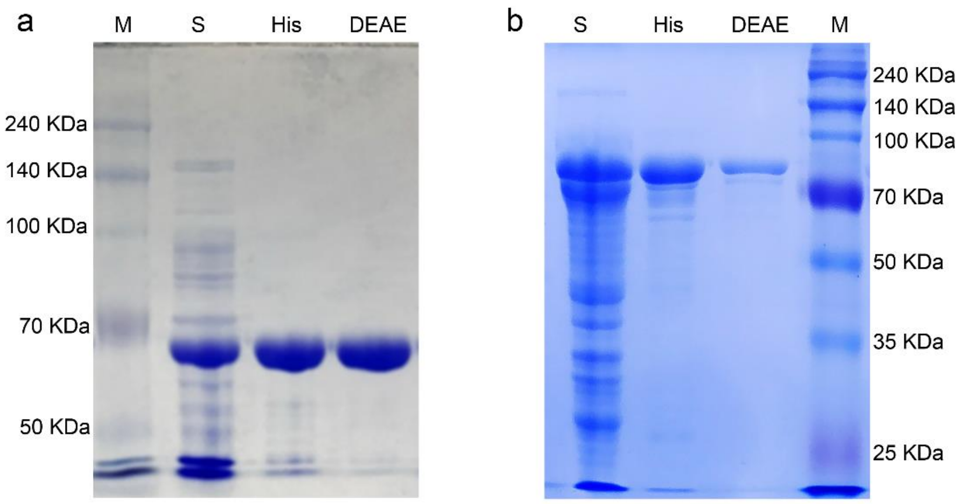

Fig. S4 SDS-PAGE analysis of recombinant MT619 (a) and C3a-MT619 (b). Lanes: M, molecular mass standard; S, crude extracts of recombinant enzymes; His, the elution fraction after Ni column purification; DEAE, the elution fraction after amylose column cellulose and DEAE column purification.

Supplementary Tables

Table S1. Bacterial strains and vectors used in the present study.

Strain or plasmid	Relevant characteristics	Source
**Strain**		
E. coli DH5α	F' Φ80lacZ• ΔM15 •*f*(lacZYA-argF)U169 deoR recA1 endA1 hsdR17(rk-, mk+) phoA supE44 thi-1 gyrA96 relA1	Enzynomics Co. Korea, Cat. #CP010
E. coli BL21 (DE3)	*E. coli* B strain with DE3, a λ prophage carrying the T7 RNA polymerase gene and lacIq<br>- Genotype : F– dcm ompT hsdS (rB- mB-) gal λ(DE3)	Enzynomics Co. Korea, Cat. #CP110
C. glutamicum ATCC 13032	Biotin-auxotrophic wild type	American Type Culture Collection (ATCC)
**Plasmid**		
pH36	KanR; *E. coli*/*C. glutamicum* shuttle vector	Construction of heat-inducible expression vector of *C. glutamicum* and *C. ammoniagenes*: fusion of lambda operator
pGEX4T-1	AmpR; Bacterial vector for expressing fusion proteins with a GST tag	Addgen Co., USA, Cat. #27458001
pET22b(+)	AmpR; pGEX derivative; Bacterial vector for expressing proteins	This study
pGEX-TS608	AmpR; pGEX derivative; containing the ts608 gene; GST tag	This study
pGEX-IB608	AmpR; pGEX derivative; containing the ib608 gene; GST tag	This study
pGEX-CV626	AmpR; pGEX derivative; containing the cv626 gene; GST tag	This study
pGEX-KA611	AmpR; pGEX derivative; containing the ka611 gene; GST tag	This study
pGEX-CS617	AmpR; pGEX derivative; containing the cs617 gene; GST tag	This study
pGEX-AS637	AmpR; pGEX derivative; containing the as637 gene; GST tag	This study
pGEX-MS614	AmpR; pGEX derivative; containing the ms614 gene; GST tag	This study
pGEX-MT619	AmpR; pGEX derivative; containing the mt619 gene; GST tag	This study
pGEX-BS642	AmpR; pGEX derivative; containing the bs642 gene; GST tag	This study
pGEX-OJ521	AmpR; pGEX derivative; containing the oj521 gene; GST tag	This study
pEX-MT619	AmpR; pEX derivative; containing the mt619 gene; His tag	This study
pEX-C3a-MT619	AmpR; pEX derivative; containing the c3a-mt619 gene; His tag	This study
pH36-C3a-MT619	KanR; pH36 derivative containing the c3a-mt619 gene; His tag	This study

Table S2. Primers used in present study

Primer	Sequence (5′ to 3′)
P1	caatttcacacaggaaacagtattcATGACCCACGCTCGCTTCC
P2	tcgagtcgacccgggaattcttagtgatggtgatggtgatgTCCGCGCTGTGCACCC
P3	CATGAATACTGTTTCCTGTG
P4	GAATTCCCGGGTCGAC
P5	caatttcacacaggaaacagtattcaTGAATTTGAAAGTGGAGTTTTACA
P6	gtcaggagcggtcaggaagcgagcgtgggtactaccacctccaccactaccacctccaccTGGCTCTTTACCCCAAACCAGGA
P7	ACCCACGCTCGCTTCCTG
P8	GGATCCCATGCTACTCCTACCAACC
P9	GCGGCCGCTGCCTG
P10	ggttggtaggagtagcatgggatccATGAATTTGAAAGTGGAGTTTTACA
P11	tgccgccaggcagcggccgcTTAATGATGGTGATGGTGATGTCCGCG

Table S3. Effects of chemical reagents and metal ions on the activity of recombinant MT619.

No.	Ions and chemical agents	Relative activity (%)
1	B-Mercaptoethanol	94 ± 2
2	$CaCl_2$	132.8 ± 2.4
3	$CoCl_2$	17.9 ± 0.5
4	$CuCl_2$	1 ± 0.2
5	DTT	79.6 ± 1.6
6	EDTA	80.9 ± 2.3
7	$HgCl_2$	-0.8 ± 0.2
8	KCl	95.3 ± 1.1
9	$MgCl_2$	112.7 ± 4.1
10	$MnCl_2$	59.9 ± 2.6
11	NaCl	128.1 ± 3.7
12	SDS	80.6 ± 6.1
13	$ZnCl_2$	3.5 ± 0.2
14	Control	100 ± 1.8
